# Ten-year survival and patient-reported outcomes of a medial unicompartmental knee arthroplasty incorporating an all-polyethylene tibial component

**DOI:** 10.1007/s00402-018-2908-y

**Published:** 2018-02-23

**Authors:** Chloe E. H. Scott, Frazer A. Wade, Deborah MacDonald, Richard W. Nutton

**Affiliations:** Department of Orthopaedics, Royal Infirmary of Edinburgh, 51 Little France Crescent, Old Dalkeith Road, Edinburgh, EH16 4SA UK

**Keywords:** Unicompartmental knee arthroplasty, Long-term survival, All-polyethylene tibia

## Abstract

**Introduction:**

Biomechanical studies have suggested that proximal tibial strain is elevated in UKAs incorporating all-polyethylene tibial components with concern that this leads to premature failure. This study reports minimum 10-year outcomes for a UKA incorporating an all-polyethylene tibial component to determine whether these concerns were realised.

**Materials and methods:**

109 fixed bearing UKAs (97 patients, mean age 68 (range 48–87), 54/97 (56%) female) with all-polyethylene tibial components were followed up for ≥ 10 years with Oxford Knee Scores, Forgotten Joint Scores and Kaplan–Meier analysis. 106/109 implants were 7 mm, 3 were 9.5 mm.

**Results:**

Ten-year survival was 85.5% (78.6–92.4 95% CI) with the end-point failure for any reason. Unexplained pain was the commonest mode of failure (6/17) followed by lateral compartment osteoarthritis (5/17) and tibial subsidence/loosening (4/17). Revision rate was highest at 2–5 years due to revisions for unexplained pain. Ten-year survival was worse in patients < 65 years old (*p* = 0.035), in those with BMI > 30 (*p* = 0.017) and in those with postoperative increases in medial tibial sclerosis (*p* < 0.001 log-rank). Implant malalignment was not significantly associated with failure. Radioisotope bone scans in 16 patients all remained “hot” at mean 6.1 years (range 2.1–11.5). Relative risk of failure in patients < 65 years was 2.9 (1.2–7.0 95% CI) and when BMI > 30 was 2.9 (1.2–6.9 95% CI). In those with intact UKAs at 10 years, mean Oxford Knee Score was 34.8 ± 10.7, Forgotten Joint Score was 37.9 ± 26.7 and 96% were satisfied with their knee.

**Conclusion:**

The high rate of early failure between 2 and 5 years in this all-polyethylene tibial component UKA did not persist in the long term. Though medial proximal tibial metabolic changes appear to persist they are not necessarily symptomatic. BMI > 30 and age < 65 years were significant risk factors for revision.

## Introduction

Joint registries consistently show higher revision rates for unicompartmental knee arthroplasties (UKAs) compared to total knee arthroplasties (TKAs) [1, 2]. Registries do not distinguish between all-polyethylene and metal-backed tibial components in fixed bearing UKAs. Some cohort studies have reported high early failure rates in all-polyethylene UKAs [3–5], but this is not consistently reported and some all-polyethylene designs perform well into the long term [6–9]. After component loosening, unexplained pain is the leading mode of failure of UKAs in the United Kingdom [10]. Elevated proximal tibial strain with repetitive microfracture and remodelling may contribute to this pain [11]. Biomechanical studies have demonstrated greater microdamage in composite bone models implanted with all-polyethylene UKA tibial components compared to metal-backed implants [12] and finite element analysis has shown elevated strain to be dependent upon all-polyethylene implant thickness [13]. Elevated proximal tibial strain under all-polyethylene UKA tibial components may cause changes in local cancellous bone architecture and predispose to early failure by tibial subsidence, aseptic loosening or ongoing pain. It is unclear whether early failures in UKAs with all-polyethylene tibial components due to unexplained pain and tibial failure continue at mid-term and long-term follow-up.

The aim of this study was to report the 10-year survival and patient-reported outcomes of a medial fixed bearing UKA incorporating an all-polyethylene component to determine

1. Whether elevated proximal tibial strain and pain resolve with time.
2. Whether an elevated short-term revision rate for “unexplained” pain persists in the longer term.

## Materials and methods

Ethical approval was obtained for this retrospective cohort study. From 2003 to 2007, 109 fixed bearing UKAs with all-polyethylene tibial components (Preservation, DePuy, Johnson & Johnson, Raynham, Massachusetts, USA) were performed in 97 consecutive patients at our institution. Mean age was 68 (median 68, range 48–87) and 54/97 (56%) patients were female. Sixty-two procedures were left sided, 106/109 (97%) utilised a 7-mm tibial component and 3/109 (3%) a 9.5-mm tibial component. Indications for surgery were isolated medial compartment degeneration with an intact ACL, fixed flexion deformity of < 10°, a correctible varus deformity of < 15°, subluxation < 1 cm and knee flexion beyond 90°. Operations were performed by two experienced consultant knee surgeons.

Medical and operation notes were reviewed for all patients. Data recorded included age, sex, weight, body mass index (BMI), indication for surgery and implants used.

Prior to surgery, all patients completed a Short-Form (SF-12) [14] Health Questionnaire (physical and mental components) and Oxford Knee Score (OKS) [15]. The OKS is a validated knee specific outcome measure of 12 questions with five possible answers giving a score from 0 to 48. Higher scores represent better function. Postoperative questionnaires including SF-12 and OKS scores were sent at 12 months. In April 2013 and again in April 2017 a similar questionnaire was sent to patients with the addition of patient satisfaction measurements, the Forgotten Joint Score (FJS) and knee specific pain questions. Patients were asked, ‘How satisfied are you with your operated knee?’ with options ‘very satisfied’, ‘satisfied’, ‘unsure’, or ‘dissatisfied’ [16]. The FJS is a validated hip/knee specific outcome measure which assesses how aware the patient is of their arthroplasty when undertaking 12 activities [17]. It is scored from 0 to 100 with 100 representing a high degree of “forgetting” their arthroplasty. Patients were asked to indicate the pain level from their knee with a visual analogue pain scale (VAS) from no pain (0) to the worst pain imaginable (100). Patients were asked if they had undergone revision or reoperation of their UKA for any reason with tick-box options. This data was correlated with the notes.

Radiographic analysis included measuring alignment [18] on preoperative and postoperative short-leg weight-bearing radiographs and examining later radiographs for evidence of implant loosening or radiographic failure. An additional measure of medial proximal tibial sclerosis (the greyscale ratio—GSR [19]) was measured using digital radiodensitometry on preoperative and follow-up radiographs at 1, 2 and 5 years. Details of this method can be found in Scott et al. [19]. This is a relative measure of sclerosis comparing the medial proximal tibial quadrant to that of the rest of the proximal tibia with a GSR > 1.0 representing relative medial sclerosis. The femorotibial angle (FTA) was measured both pre- and postoperatively in addition to postoperative coronal and sagittal implant alignment [18]. Patient imaging histories were examined to identify radioisotope bone scans. Where performed the indication for radioisotope bone scanning was recorded and the results examined.

### Statistical analysis

Statistical analysis was performed using Statistical Package for Social Sciences version 21.0 (SPSS Inc., Chicago, Illinois). In patients who had undergone bilateral UKA, PROMs pertaining to the second knee were excluded to avoid bias. Univariate analysis was performed using parametric (Student’s *T* test: paired and unpaired) and non-parametric (Mann–Whitney *U* test) tests as appropriate to assess continuous variables for significant differences. Nominal categorical variables were assessed using a Chi-square or Fisher’s exact test. Pearson’s correlation was used to assess the relationship between linear variables. A *p* value of < 0.05 was considered statistically significant. Survival analysis was undertaken with life-tables and Kaplan–Meier analysis. The end points used were failure for any reason and revision to TKA.

## Results

**Fig. 1:**
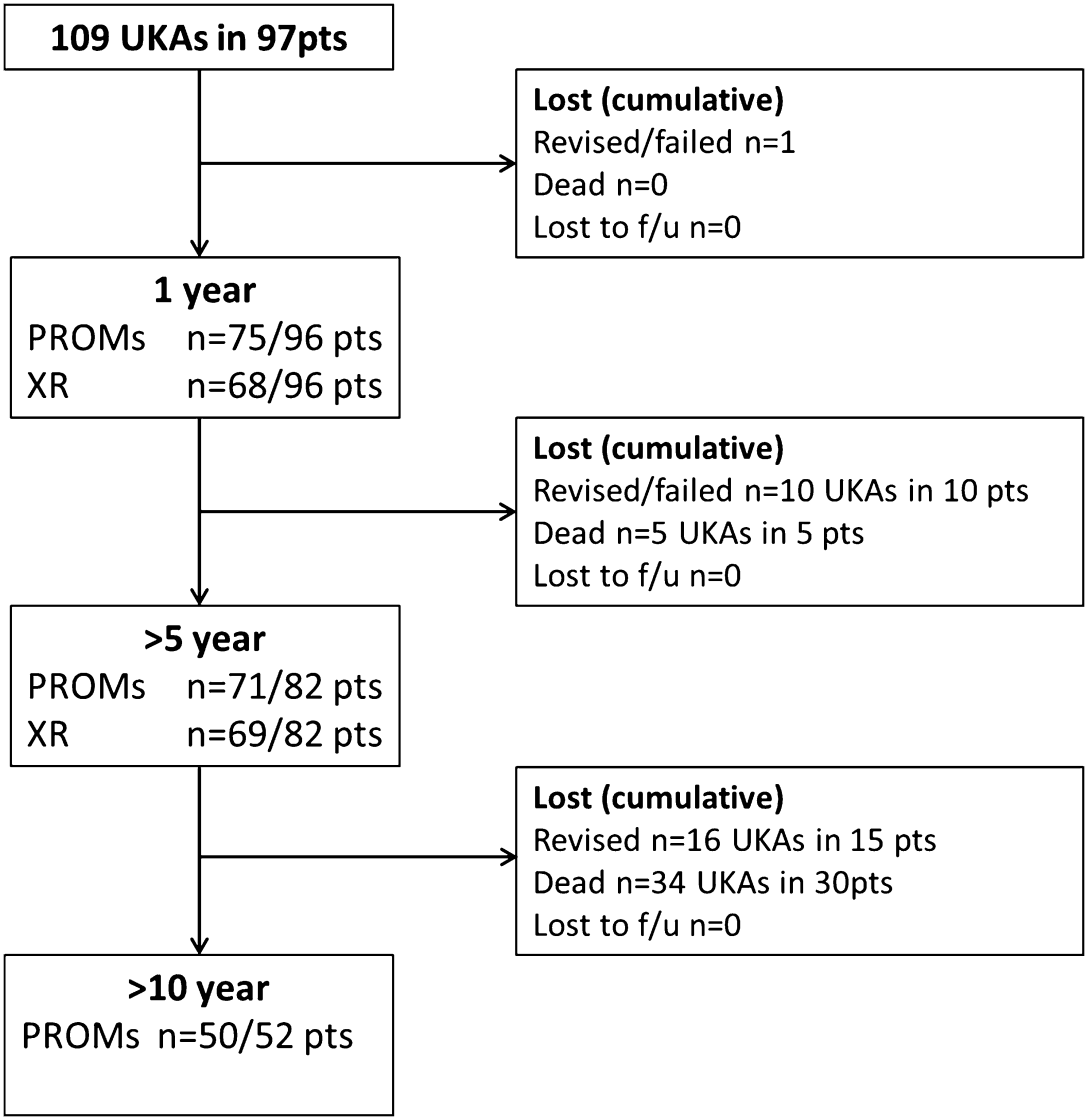
Consort diagram of UKA cohort

During the study period 109 fixed bearing UKAs with all-polyethylene tibial components were implanted in 97 patients (Fig. 1). Preoperative characteristics are given in Table 1. Over the study period 28 patients with 32/109 UKAs died (29%) and a further 16/109 (15%) were revised. Mean length of follow-up was 11.4 years (1.0 SD, 10.0–13.2) and no patients were lost to follow-up.

**Table 1:** Preoperative patient characteristics

	Mean (SD), number [%]	Range
Age	68 (8.9)	48–87
Female	54/97 [56]	
BMI	28.8 (4.6)	20–42
Weight	79.1 (14.9)	48–110
Femorotibial angle (deg)	181.6 (2.6)	175–191
Indication		
OA	107/109 [98]	
AVN	2/109 [2]	
PROMs		
SF-12		
PCS	31.2 (7.1)	17–48
MCS	50.7 (11.5)	19–67
OKS	20.2 (5.9)	8–34

### Survival analysis

**Fig. 2:**
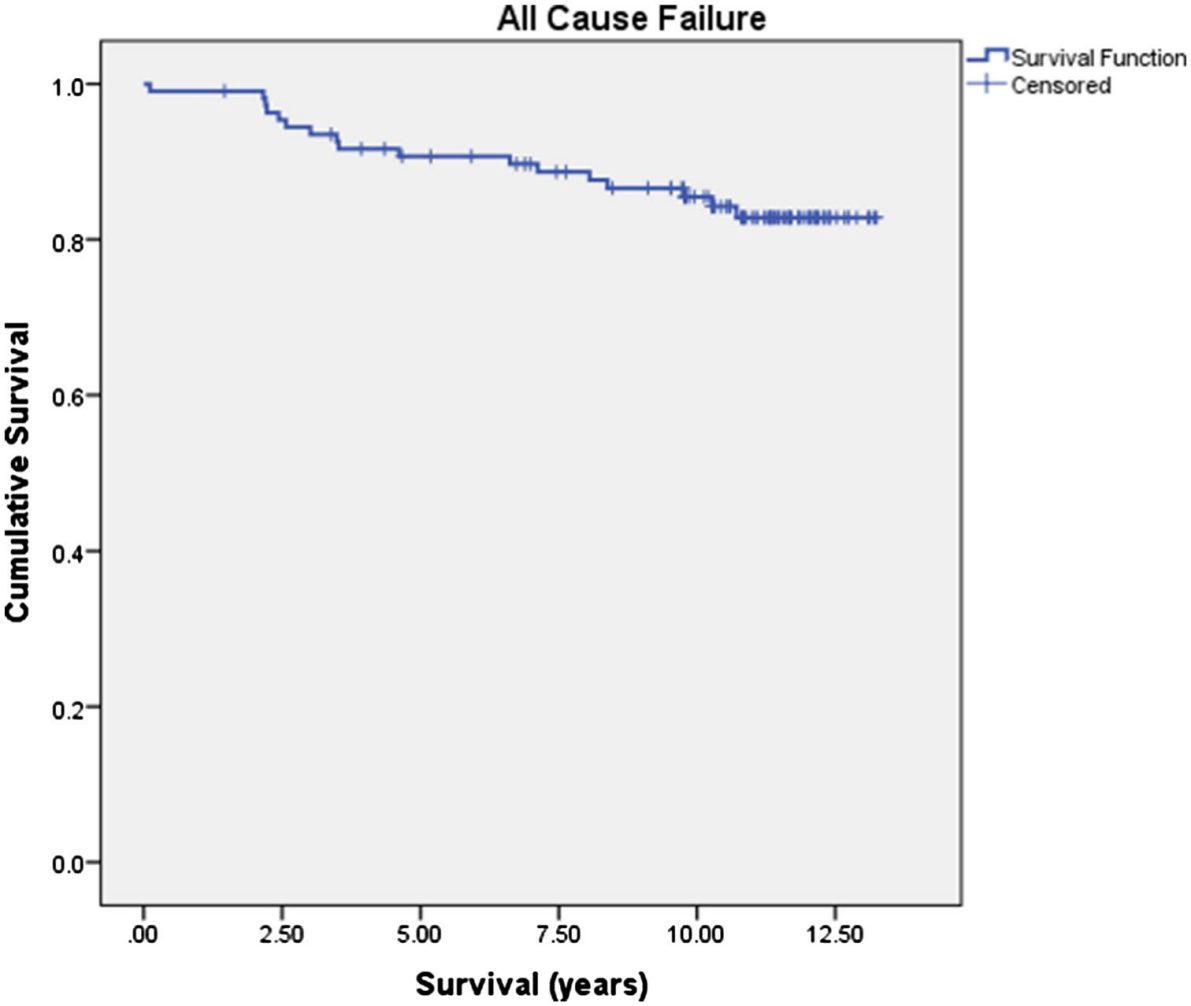
Kaplan–Meier survival analysis at 10 years with all failures as the end point

The life table for all failures is given in Table 2. Over the study period, 17 UKAs failed and 16 were revised (Table 3). One case of failure by tibial subsidence at 9 years has not been revised due to poor medical health. All failures incorporated a 7-mm tibial component. Revisions for unexplained pain were performed earlier (mean 3.5 years, range, 2.2–6.6) than revisions for other causes (mean 5.6 years, range, 0.2–10.7), though this was not statistically significant (*p* = 0.350, Mann–Whitney *U* test). Kaplan–Meier analysis demonstrated 10-year survival of 86.6% (80.1–93.1% 95% CI) with revision as an end point. Including the additional unrevised case of tibial subsidence gave 10-year survival of 85.5% (78.6–92.4 95% CI) with failure for any reason as an end point (Fig. 2). Excluding revisions for unexplained pain, which could be considered as discretionary, gave a 10-year survival of 90.8% (85.1–96.5 95% CI).

**Table 2:** Life table for all failures

Interval	*N*	Failures	Withdrawals	At risk	Failure rate (%)	Cumulative survival	95% CI
0–1	109	1	0	109	1	99.1	97.3–100
1–2	108	0	1	107.5	0	99.1	97.3–100
2–3	107	5	0	107	5	94.5	90.2–98.8
3–4	102	3	2	101	3	91.6	86.4–96.8
4–5	97	1	2	96	1	90.7	85.2–96.2
5–6	94	0	2	93	0	90.7	85.2–96.2
6–7	92	1	3	90.5	1	89.7	83.9–95.5
7–8	88	1	2	87	1	88.7	82.6–94.8
8–9	85	2	1	84.5	2	86.6	80.0–93.2
9–10	82	1	10	77	1	85.4	78.6–92.2
10–11	71	2	17	62.5	3	82.7	75.0–90.3
11–12	52	0	27	38.5	0	82.7	75.0–90.3
> 12	29	0	25	14.5	0	82.7	75.0–90.3

**Table 3:** Failed UKAs

Sex	Age	BMI	Survival (years)	Mode of failure	Malalignment?
M	66	29	0.2	Tibial subsidence	Varus tibia 6°
F	71	30	2.2	Aseptic loosening femur	Varus tibia 6°
F	78	26	2.2	Lateral OA	Flexed femur 22°
F	55	34	2.25	Pain	
M	61	32	2.4	Pain	
M	61	31	2.6	Periprosthetic fracture	
M	65	29	3.0	Pain	Varus tibia 6°
F	60	31	3.5	Pain	
M	65	31	3.5	Pain	
F	70	31	4.6	Lateral OA	Varus femur 6°
M	60	39	6.6	Pain	
F	53	32	7.1	Lateral OA	Reverse tibial slope 3°
F^a^	62	37	8.1	Aseptic loosening femur & tibia	
F	61	21	8.4	Lateral OA	Varus tibia 5°
F	55	30	10.3	Lateral OA	Varus tibia 8°
F^a^	61	37	10.7	Tibial subsidence	

^a^Denotes the same patient requiring revision of bilateral UKAs

**Fig. 3:**
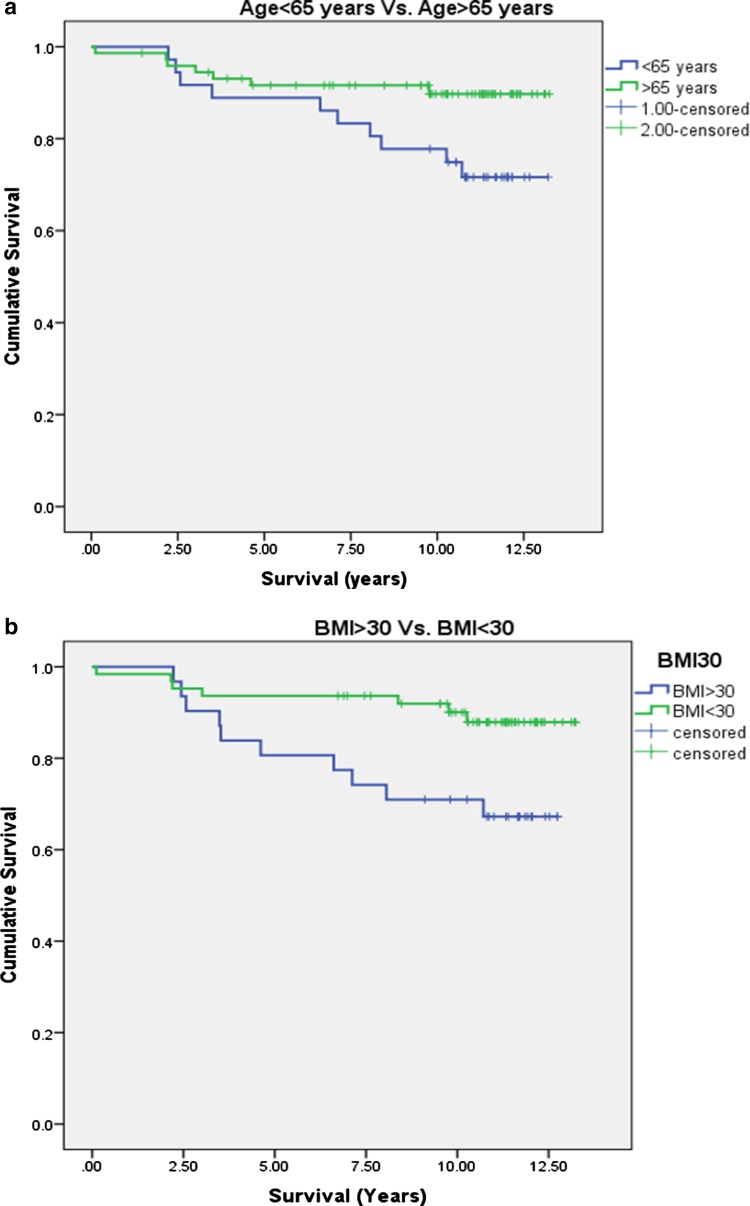

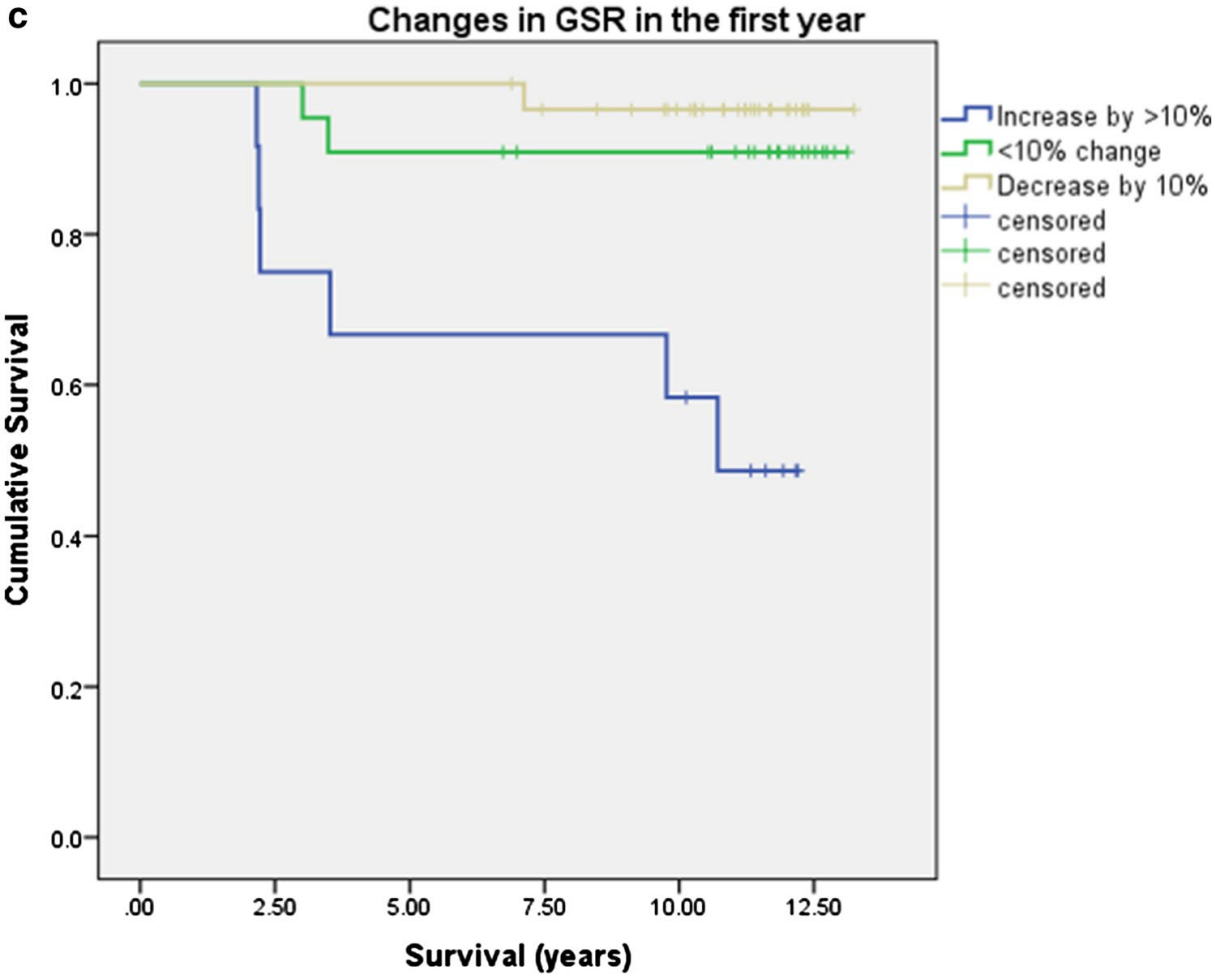
Kaplan–Meier analysis of 10-year survival with subgroup analysis for **a** age above and below 65 years, **b** BMI above and below 30 and **c** change in GSR over the first year

**Fig. 4:**
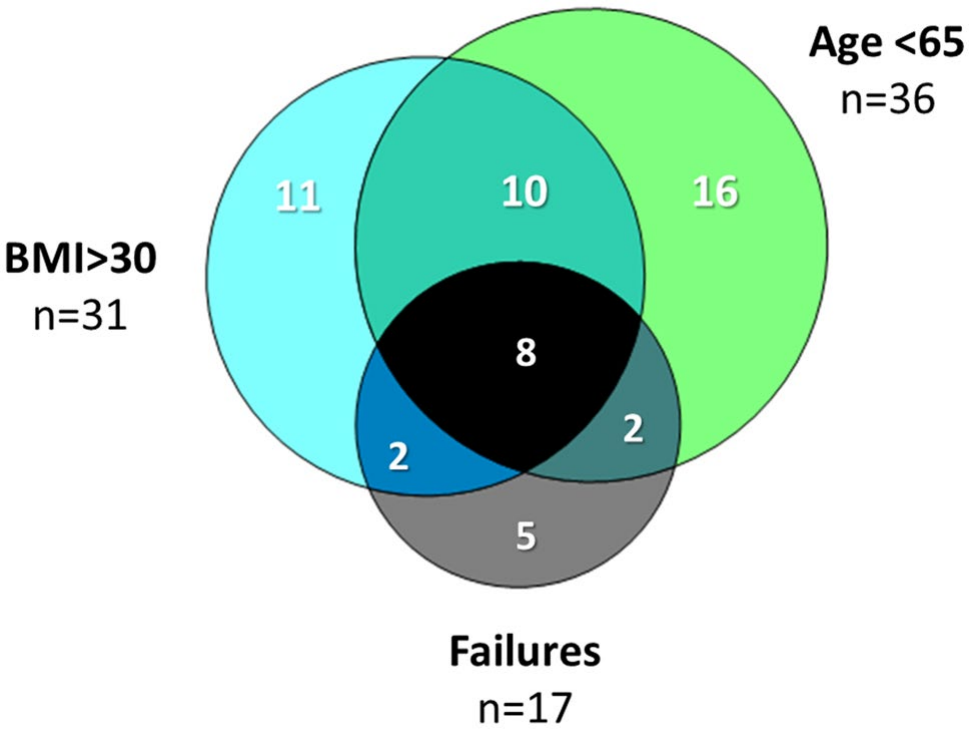
Venn diagram showing the relationship between age < 65, BMI > 30 and UKA failures

Characteristics of patients with intact and failed UKAs are given in Table 4. With failure for any reason as an end point, 10-year survival was significantly inferior in patients under 65 years of age, patients with BMI > 30 and in those whose GSR increased by greater than 10% in the first year (Table 5; Fig. 3). BMI was significantly higher in patients < 65 years compared to those ≥ 65 (30.9 ± 5.4 vs 27.6 ± 3.6, *p* = 0.001, unpaired *T* test), and age was significantly younger in patients with BMI > 30 compared to those with BMI < 30 (62.8 ± 6.7 vs 70.1 ± 8.7, *p* < 0.001, unpaired *T* test). Figure 4 demonstrates the relationship between age, BMI and failures. Of 18 patients both < 65 years old and with BMI > 30, 8 (44%) failed requiring revision (*p* = 0.001, Chi-squared).

**Table 4:** Characteristics of intact and failed UKAs at 10 years

	Intact (*n* = 92)	Failed (*n* = 17)	*p* value	95% CI
Demographics				
Age	69.3 (9.1)	63.9 (6.6)	0.019*	− 10.1 to − 0.91
Female	49 [54]	11 [65]	0.408^	
BMI	28.2 (4.5)	31.1 (4.3)	0.020*	0.45 to 5.20
Weight	78.1 (14.9)	82.5 (14.5)	0.264*	− 3.42 to 12.35
GSR				
Preop	1.11 (0.18)	1.04 (0.13)	0.286*	− 0.2 to 0.06
1 year	1.03 (0.2)	1.15 (0.21)	0.077*	− 0.01 to 0.25
5 years	1.00 (0.12)	1.13 (0.18)	0.005*	0.04 to 0.20
GSR year 1 change				
Absolute change	− 0.08 (0.18)	0.15 (0.16)	0.001*	0.10 to 0.36
> 10% increase	6 [10]	6 [67]	< 0.001^	
< 10% change	20 [36]	2 [22]		
> 10% decrease	29 [53]	1 [11]		
Alignment				
Femorotibial angle	177.7 (2.9)	177.6 (4.3)	0.895*	− 2.3 to 1.5
Medial proximal tibial angle	85.8 (2.4)	86.7 (2.7)	0.235*	− 1.1 to 2.2
Posterior tibial slope	2.5 (3.1)	1.7 (2.5)	0.397*	− 1.6 to 1.2
Femur coronal (degrees of valgus)	4.5 (4.9)	2.9 (5.0)	0.250*	− 1.1 to 4.9
Femoral flexion	− 1.0 (7.0)	− 4.5 (6.4)	0.103*	− 7.7 to 0.7
PROMs				
PCS				
Preop	31.5 (7.0)	26.9 (7.7)	0.212*	− 12 to 2.7
1 year	40.0 (11.3)	41.3 (12.4)	0.850*	− 12.6 to 15.2
5 year	40.9 (11.2)	37.4 (14.4)	0.520*	− 14 to 7.2
10 year	40.7 (13.6)	32.2 (7.1)	0.039*	− 16.7 to − 0.4
MCS				
Preop	50.6 (11.6)	52.0 (12.2)	0.830 т	
1 year	50.7 (10.6)	54.4 (14.4)	0.682 т	
5 year	49.6 (10.9)	45.5 (13.0)	0.522 т	
10 year	49.2 (15.3)	43.8 (12.8)	0.161 т	
OKS				
Preop	20.3 (6.0)	18.3 (4.9)	0.698 т	
1 year	33.9 (9.4)	29.3 (11.1)	0.525 т	
5 year	36.1 (10.1)	26.4 (13.7)	0.104 т	
10 year	34.1 (10.9)	26.6 (8.4)	0.014 т	

*Unpaired *T* test, ^ Chi-squared, ^т^Mann–Whitney *U* test

**Table 5:** Ten-year Kaplan–Meier survival analysis (end point = any cause failure) by subgroup

Variable	Division	*N*	Survival (%)	95% CI	*p* value
Gender	Male	48	87.3	77.9–96.7	0.501*
	Female	61	84.3	74.9–93.7	
Age	< 65 years	36	77.8	57.4–98.2	0.035*
	≥ 65 years	73	89.7	82.4–97.0	
BMI	≤ 30	63	90.0	82.3–97.6	0.017*
	> 30	31	71.0	54.9–87.1	
GSR change in year 1	Increase by > 10%	12	58.3	30.5–86.1	< 0.001*
	Change < 10%	22	90.9	78.9–100	
	Decrease by > 10%	30	96.6	89.9–100	

*Log-rank

Relative risk of failure in patients under 65 was 2.9 (1.2–7.0 95% CI) times that of patients over 65 years. Relative risk of failure in patients with BMI > 30 was 2.9 (1.2–6.9 95% CI) times that of patients with BMI ≤ 30. Relative risk of failure in patients < 65 with BMI > 30 was 3.64 (1.7–7.8 95% CI). Ten of the 17 UKA failures displayed tibial-sided failure (tibial subsidence/loosening, fracture or unexplained pain). Ten-year survival functions for tibial-sided failure are shown in Table 6. Tibial survival was again significantly inferior in patients under 65 years of age, those with BMI > 30 and in those with medial sclerosis with an increase in GSR of > 10%.

**Table 6:** Ten-year Kaplan–Meier survival analysis (end point = tibial-sided failures only) by subgroup

Variable	Division	*N*	Survival (%)	95%CI	*p* value
Gender	Male	48	87.3	77.9–96.7	0.458*
	Female	61	92.5	85.4–99.6	
Age	< 65 years	36	83.2	71.0–95.4	0.040*
	≥ 65 years	73	93.8	87.9–99.7	
BMI	≤ 30	63	94.8	89.1–100	0.004*
	> 30	31	77.0	62.1–91.9	
GSR change in year 1	Increase by > 10%	12	70.0	38.4–100	0.001*
	Change < 10%	22	90.9	78.9–100	
	Decrease by > 10%	30	100		

*Log-rank

Of six patients who were revised and had increases of > 10% in GSR, two had pain and 2 tibial subsidence as the mode of failure.

### PROMs

Long-term PROMs were available for 67/92 (73%) patients alive with intact UKAs at 5 years and 57/58 (98%) patients alive with intact UKAs at 10 years. At 10 years, 96% with intact UKAs were satisfied or very satisfied with their knee. This was significantly greater than those satisfied at 5 years (81.5%, *p* = 0.007, Chi-squared), despite a decline in OKS from 5 to 10 years. This may reflect revision of a further 6 UKAs between 5 and 10 years. The mean Forgotten Joint Score at 10 years was 37.9 ± 26.7 (range 0–80).

The decline in mean OKS from 5 to 10 years was not significant (*p* = 0.388 paired *T* test, − 1.5 to 3.8 95% CI) and the improvement in OKS from preoperative levels remained at 10 years (*p* < 0.001 paired *T* test, − 15.8 to − 8.1 95% CI). Following revision to TKA, patients revised for pain had significantly worse OKSs than patients revised for other reasons (21.8 ± 8.6 vs 31.3 ± 5.2, *p* = 0.043 unpaired *T* test, 0.3–18.6 95% CI). VAS pain scores increased from a median of 7 (mean 20, 0–99) at 5 years to 20 (mean 27, 0–85) at 10 years, though this was not significant (*p* = 0.309, Wilcoxon rank).

Age did not correlate significantly with OKS or with SF-12 physical component scores (PCS) or mental component scores (MCS) at any time point. There was no significant difference in OKS between patients older and younger than 65 (32.9 ± 11.4 vs 32.7 ± 10.3, *p* = 0.923 unpaired *T* test, − 5.5 to 5.0 95% CI) or between those with BMIs greater than or less than 30 (33.7 ± 10.3 vs 32.4 ± 11.1, *p* = 0.628 unpaired *T* test, − 4.2 to 6.9 95% CI) at 10 years, or at any other timepoint.

### Radiographic outcomes

Component alignments are detailed in Table 4. There were no significant differences in tibial or femoral alignment between UKAs which went on to fail for any reason (Table 4) or for tibial-sided failure. Over or underhang was not significantly associated with all-cause failure (*p* = 0.731, unpaired *T* test) or tibial-sided failure (*p* = 0.61, unpaired *T* test). Alignment outliers (> 5° varus, reverse tibial slope, excessive PTS > 10°, femoral extension > 10° or flexion of > 10°, and femoral valgus > 10° or varus) were not associated with failure (*p* = 0.534, Chi-squared). Tibial (*p* = 0.345, Chi-squared) or femoral (*p* = 0.299, Chi-squared) malalignment was not significantly associated with failure.

Absolute GSR was significantly greater at 5 years in patients whose UKA went on to fail (Table 4) and implant survival was significantly worse in patients whose GSR had increased by > 10% (Fig. 3c; Tables 5, 6).

Sixteen UKAs in 12 patients underwent radioisotope bone scanning at mean 6.1 ± 3.3 years (2.1–11.5 years). Bone scanning was performed for reasons unrelated to the UKA in 4 cases. All UKAs that underwent radioisotope bone scanning showed well localised increased uptake in the medial proximal tibia under the tibial component. Seven went on to be revised: 4 for “unexplained” pain; 2 for tibial subsidence/loosening; and one for lateral OA. Of the 9 UKAs with hot bone scans who were not revised, 50% were satisfied with their UKA at 5 years, but 100% were satisfied at 10 years. One reported medial pain at 5 years and one at 10 years.

In addition to the 17 failures already discussed, there were no other radiographic failures on review at a mean of 7.8 ± 2.7 years postoperatively.

## Discussion

The 10-year survival of this UKA incorporating an all-polyethylene tibial component was found to be 85.5% (78.6–92.4 95% CI) with failure for any reason as an end point. Unexplained pain was the commonest mode of failure (6/17, 35%) followed by osteoarthritis progression (5/17, 29%) and tibial subsidence/loosening (4/17, 24%). Survival was inferior in patients < 65 years, in those with a BMI > 30 and in those whose medial tibial bone density increased postoperatively. This was the case for all failures and for tibial-sided failures including pain. It is unclear whether age < 65 or BMI > 30 is a more important risk factor for failure, but 44% (8/18) of patients who were both < 65 years old and had a BMI > 30 went on to fail. Revisions for unexplained pain were performed earlier than revisions for other reasons. In those with intact UKAs satisfaction at 10 years was high at 96% and improvements in OKS were maintained, despite slight deteriorations in pain levels as measured using VAS pain scores.

Our 10-year survivorship is consistent with UKA joint registry data [1, 2]. Rates of aseptic loosening, progression of osteoarthritis and revision for unexplained pain are similar to those reported in the literature [10, 20]. Joint registries do not differentiate between all-polyethylene and metal-backed UKAs. In the literature there are reports of both favourable [6–9] and concerning survivorship of medial UKAs incorporating all-polyethylene tibial components [3–5]. The Norwegian Arthroplasty Register [21] reports significantly higher rates of revision at 5 years in 2 all-polyethylene designs compared to 3 metal-backed mobile bearing devices. Saenze et al. [3] report a failure rate of 11% (16/144) at mean 36 months with tibial failures in 12/16 (75%). Mariani et al. [22] report a 38% revision rate (15/39) at 12 months with femoral loosening in all failures. Hamilton et al. [23] reported 9/221 (4%) revisions at 1–26 months, 4/9 for tibial loosening/collapse. These implants had minimum polyethylene (PE) thickness 7.5 mm. In contrast, the St Georg Sled (Waldemar Link, Hamburg, Germany), an all-polyethylene implant with minimum polyethylene thickness 9 mm reports good long-term survival of 90–92% at 10–15 years [6, 7] with no revisions for ongoing pain and no early failures.

Concerns have thus been raised regarding the incidence of tibial loosening/subsidence in all-polyethylene tibial components [4, 24], and the role of implant stiffness in unexplained pain [12, 19, 25]. Such variable survival rates from 56% at 7 years (32–75 95% CI) [4] to 87.6% at 10 years [9] in all-polyethylene designs suggest that not all all-polyethylene designs are equal, and this may reflect component thickness [13]. Components of 6 mm thickness have been significantly associated with early clinical failure [26], increased wear and osteolysis [27]. Pathological cancellous bone overload and tibial subsidence may affect thinner implants more so than thicker implants. Finite element analysis has shown that increasing all-polyethylene thickness to 10 mm still under-performs in terms of proximal tibial strain compared to metal-backed implants [13] with the added cost of increased tibial resection and associated reduction in cancellous bone strength [28].

Contrary to Cavaignac et al. [29] and Zengerink et al. [30] who reported no effect of obesity on revision rates in 212 and 147 patients, respectively, and consistent with a theory of bone overload and pain, we found significantly poorer survival in patients with BMI > 30. Increased risk of revision in patients under 65 years concurs with Dyrhovden et al. [31] who examined 725 revisions out of 7648 UKAs from the Norwegian Joint Registry. The combination of these two variables seemed particularly relevant here with 44% of those < 65 years with a BMI > 30 failing.

We have previously published a digital radiodensitometry technique in this UKA cohort to investigate the relative bone density of the medial proximal tibial [19]. A proxy measure of bone mineral density—the grey scale ratio (GSR)—was developed with a value of > 1.0 reflecting relative medial sclerosis [19]. Consistent with other reports of BMD following UKA [32], we found that on average GSR decreases in the first year following medial UKA and then remains static. Increases in GSR were associated with worse OKS and pain. The present study confirms that an increase in relative sclerosis is also associated with worse 10-year survival. Similarly, Jacobs et al. [33] demonstrated that preoperative bone marrow oedema did not resolve following UKA, and that when present postoperatively was associated with worse pain. Both phenomena may reflect ongoing medial strain and adaptive remodelling. This is supported by persistently “hot” radioisotope bone scans here.

It has been previously suggested that proximal tibial adaptive remodelling after UKA stabilises at 2 years with resolution of pain at that stage [11]. This is not supported by our results. When performed, bone scans demonstrated persistently high medial tibial uptake which did not resolve by 2 years and revisions for unexplained pain were performed up to 7 years, consistent with National Joint Registry data [10, 34]. Thirty percent of patients reported ongoing medial pain at > 5 years and 25% at 10 years. Persistently “hot” bone scans may reflect persistent bone marrow oedema and pain [33], but here not all those with “hot” bone scans reported ongoing pain. The role of bone scintigraphy in the investigation of painful UKAs remains unsupported [35]. Revision to TKA for pain had a significantly worse OKS outcome compared to revisions for other reasons and this is consistent with Kerens et al. [36]. Though suboptimal implant alignment was not statistically predictive of failure, it may have played a role in up to 50% of failed UKAs.

Limitations of this study include the sample size and its retrospective nature. Implant alignment was measured on short-leg radiographs, not hip–knee–ankle radiographs, and may be less accurate. The Preservation UKA is no longer available and has since been redesigned as the Sigma Partial Knee (DePuy Synthes, Johnson & Johnson, Raynham, Massachusetts, USA) with the addition of a peg to the keel of the tibia and an additional femoral component peg. The minimal thickness of all-polyethylene bearing has been increased to 8 mm. No patients were lost to follow-up in this 10-year study the results of which highlight issues specific to all-polyethylene UKA components which should be considered in implant selection.

## Conclusion

This UKA incorporating an all-polyethylene tibial component was associated with a high rate of early failure between 2 and 5 years, predominantly due to unexplained pain and tibial-sided failure. Though metabolic changes in the medial proximal tibia appear to persist into the long term on bone scans, this is not necessarily symptomatic. The elevated rate of early revision did not persist with 10-year survival for all-cause failure of 85.5% (78.6–92.4 95% CI). Patients < 65 years of age and those with BMI > 30 kg/m^2^ displayed significantly worse 10-year survival with this implant and 44% of patients with both risk factors went on to fail.
